# Fluorescence *in situ *hybridization (FISH) analysis of primary ocular adnexal MALT lymphoma

**DOI:** 10.1186/1471-2407-6-249

**Published:** 2006-10-20

**Authors:** Kazuki Tanimoto, Naohiro Sekiguchi, Yukiko Yokota, Akihiro Kaneko, Takashi Watanabe, Akiko Miyagi Maeshima, Yoshihiro Matsuno, Mine Harada, Kensei Tobinai, Yukio Kobayashi

**Affiliations:** 1 Hematology and Stem Cell Transplantation Division, National Cancer Center Hospital, 5-1-1 Tsukiji, Chuo-ku, Tokyo, Japan; 2 Ophthalmology Division, National Cancer Center Hospital, 5-1-1 Tsukiji, Chuo-ku, Tokyo, Japan; 3 Pathology Division, National Cancer Center Hospital and Research Institute, 5-1-1 Tsukiji, Chuo-ku, Tokyo, Japan; 4 Medicine and Biosystemic Science, Kyushu University Graduate School of Medical Sciences, 3-1-1 Maidashi, Higashi-ku, Fukuoka, Japan

## Abstract

**Background:**

It remains unknown whether primary ocular adnexal extranodal marginal zone B-cell lymphoma of mucosa-associated lymphoid tissue (MALT lymphoma) is a homogeneous entity, as there are few reports of the results of cytogenetic or molecular analyses of these tumors.

**Methods:**

We performed interphase fluorescence *in situ *hybridization (FISH) analysis to detect translocations and aneuploidy in 34 cases of primary ocular adnexal MALT lymphoma, and reviewed the histopathological findings. Correlations between the results of FISH analysis, the histopathological features and the clinical data were also analyzed.

**Results:**

Among the 34 cases, FISH analysis revealed t(14;18)(q32;q21) in one case, trisomy 3 in 21 cases (62%), and trisomy 18 in 16 cases (47%). The cases with trisomy 18 had significantly more prominent lymphoepithelial lesions (LELs) and less nodularity in the tumors. In regard to the clinical correlations, tumors with trisomy 18 were observed predominantly in females and younger patients; also, in the majority of the cases, the tumor was of conjunctival origin. All the cases with recurrence showed trisomy 18 in the tumor.

**Conclusion:**

Primary ocular adnexal MALT lymphoma is a significantly heterogeneous entity. Cases with trisomy 18 may have unique clinicopathological features.

## Background

Since the first description by Isaacson and Wright in 1983, MALT lymphoma has been recognized as a distinct entity of low-grade B-cell lymphoma; it is described as a separate entity in the revised European-American lymphoma (REAL) classification and also in the more recent classification by the World Health Organization (WHO) [1-3].

Various cytogenetic changes, such as t(11;18)(q21;q21), and t(1;14)(p22;q32) and trisomy 3, 7, 12 and 18, have been detected as recurrent chromosomal aberrations in this tumor [4-8]. Recently, t(14;18)(q32;q21), which is derived from fusion of the immunoglobulin heavy chain (IgH) gene with the MALT lymphoma-associated translocation (MALT1) gene has been described [9]. While it is difficult by standard karyotypic analysis to distinguish this translocation from that in follicular lymphoma which is derived from fusion of the IgH gene with the bcl-2 gene, FISH analysis is useful for the differentiation. The histopathological findings of MALT lymphoma have also been reported to be heterogeneous [2,3]. The cells comprising the tumor may be either centrocyte-like cells, cells resembling monocytoid B cells, small lymphocytes or scattered immunoblasts and centroblast-like cells. There may be various degrees of plasma cell differentiation.

Malignant lymphomas arising from the ocular adnexa account for about 8% of all extranodal lymphomas [10]. They have been recognized as low-grade B-cell lymphomas which usually remain localized and follow an indolent clinical course [11,12]. Several reports have suggested that the majority of lymphomas arising from the ocular adnexa are MALT lymphomas [13-17]. As heterogeneity among MALT lymphomas are well known, it would also be reasonable to expect heterogeneity in the case of ocular adnexal MALT lymphomas; however, the significance of the cytogenetic and histopathological differences among the tumors is still not known. In this study, we conducted a retrospective analysis of a large number of cases with ocular adnexal MALT lymphoma to determine the nature and distribution of chromosomal aberrations as evaluated by FISH and the tumor grade as evaluated by histopathological examination, and also attempted to determine the relationships between the clinical features, chromosomal aberrations and the histopathological findings.

## Methods

### Patients' samples

Archived samples of primary ocular adnexal MALT lymphoma at our institute between 1995 and 2003 were utilized for this study. The diagnosis of MALT lymphoma was confirmed by histopathological examination of either biopsy specimens or surgically resected specimens of the primary ocular adnexal lesions. In all the cases, the diagnosis was reviewed by two hematopathologists (YM and AAM) according to the REAL classification or the WHO classification. Samples for the FISH analysis were obtained from the same primary lesions in the ocular adnexa.

This study was approved by the institutional review board of National Cancer Center (16–7).

### Clinical features

The clinical data were obtained from the medical records. The sex, age, clinical stage, site of involvement, performance status (PS) according to the Eastern Cooperative Oncology Group scale, the serum lactate dehydrogenase (LDH) value at the time of initial presentation, and the initial therapy were recorded. The anatomic site of involvement was determined by the system reported by Knowles et al. [18]. We specified lachrymal gland origin when the gland structure could be clearly recognized in the histopathological specimens. Chest X-ray, computed tomography of the head/eye, neck, chest, abdomen and pelvis, gallium scintigraphy, bone marrow and peripheral blood examinations were performed to determine the clinical stage. The clinical stages were determined according to the Ann Arbor staging classifications. Disease recurrence was defined as an increase in the tumor size as detected by either visual inspection or by imaging studies, or appearance of new tumor lesions at distant sites.

### FISH analysis

The tissue FISH procedure was performed in formalin-fixed paraffin sections in accordance with the procedure described by Sekiguchi et al. [19]. We applied six kinds of probes for the FISH analysis. All of the probes were purchased from the same manufacturer (Vysis, Downers Grove, IL, USA). To detect t(14;18)(q32;q21), we used the LSI IGH/MALT1 Dual Color, Dual Fusion Translocation Probe. To detect t (11;18)(q21;q21), we used the LSI API2/MALT1 Dual Color, Dual Fusion Translocation Probe. All the specimens were examined using the IGH/MALT1 probe to detect t(14;18)(q32;q21). In cases in which extra MALT1 signals were detected, we used the API2/MALT1 probe to detect t(11;18)(q21;q21). To detect amplification or deletion of chromosomes 3,7,12 and 18, we used the CEP 3, 7, 12 and 18 Spectrum Orange or Green Probes which hybridize to the centrometric region of the chromosomes.

We also analyzed 4 reactive lymphomatous lesions of the ocular adnexa as a negative control and the threshold was determined for each probe by counting the number of signals or fusions in one nucleus per 100–200 cells. The signal frequency thresholds were determined as the mean plus 3 standard deviations. Each specimen was examined independently by three investigators (YK, YY, and KT). The number of signals or fusions in one nucleus was counted in 200 cells, and when the results were judged to be the same, the specimen was considered as being successfully stained and included in this study.

### Histopathological review

Each hematoxylin eosin (HE)-stained specimen was histologically reviewed by two of us (KT and YM). In addition to histopathology, the paraffin-embedded sections were also subjected to immunostaining for CD3 by the standard avidin – biotin method using monoclonal anti-CD3 antibody (PS1, Novocastra, Newcastle-upon-Tyne, UK) as the primary antibody. Immunophenotypic analysis revealed that the tumor cells of MALT lymphoma were positive for CD20 and negative for CD5, CD10 and cyclin D1. Frequently but not universally observed morphological features of primary ocular adnexal MALT lymphomas were examined for and categorized as follows: the finding of an abundance of plasma cells, monocytoid B cells and reactive CD3-positive T cells in none of the areas of the tumor was denoted as negative (-), in less than 30% of the tumor area was denoted as focally positive (+), in 30–60% of the tumor area was denoted as positive (2+), and in 60% or more of the tumor area was denoted as strongly positive (3+). The finding of residual reactive germinal centers, large lymphoid cells, polykaryocytes, brown-pigment-laden histiocytes, mitotic cells, lymphoepithelial lesions (LELs) and Dutcher bodies was categorized as negative (-), focally positive (+), or positive (2+) when it was absent, present but inconspicuous, or very conspicuous, respectively. The degree of nodularity was classified as negative (-) in cases without discernible vague nodularity, focally positive (+) in cases with vague nodularity, and positive (2+) in cases showing definite nodularity.

### Statistical analysis

Statistical analysis was performed using the SPSS software for Windows, version 13.0. The correlations between the clinical features, results of FISH analysis and the morphological features were analyzed by Fisher's exact test. Survival was censored at the time of the last documented follow-up date. The overall survival period and time to recurrence were calculated using the Kaplan-Meier method and compared by the log-rank test.

## Results

### Patient characteristics and outcomes

The characteristics of the patients are shown in Table 1. The median age was 57 years (range, 15 to 90 years), and there were 23 males and 11 females. There were three age peaks; 8 patients who were less than 40 years of age, 12 patients who were 50 years of age and 14 patients who were more than 60 years of age.

The most frequently involved site was the orbit (62%). In 26 patients (76%), the disease was unilateral, whereas in eight patients (24%) it was bilateral. Almost all patients had localized disease and a serum LDH in the normal range. None of the patients had any manifestations of autoimmune diseases. The initial therapies varied among the patients: 21 patients (66%) received radiation only, three (9%) received chemotherapy, three (9%) received chemotherapy combined with radiation therapy and 5 (16%) were observed under a watch-and-wait policy. Two patients were lost to follow-up after the diagnosis. After a median follow-up duration of 31 months (range, 8 to 118 months), all 32 patients were alive and the disease recurred in five patients. There were no significant differences in terms of the sex or age of the patients, site of involvement, laterality, initial therapy, stage, or the serum LDH level at the time of initial presentation (data not shown).

### FISH analysis and its relationship to clinical factors

The signal frequency thresholds were determined as the mean plus 3 standard deviations (SD). The mean values of more than two signals for the CEP 3, 7 and 18 probes were 2.50%, 0.67% and 1.75%. The thresholds of the CEP 3, 7 and 18 probe signals were 4.23%, 2.40%, and 3.25%. IgH/MALT1 and API2/MALT1 fusions and more than two signals for CEP 12 probe were not observed for any of the 4 control samples. Based on these results, we decided to set a cut-off value of 5% for all the probes to reduce the incidence of false-positive results.

**Figure 1:**
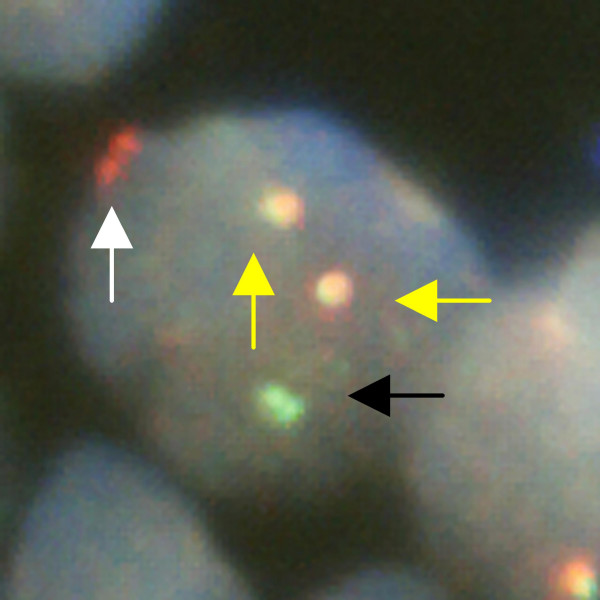
Representative result of fluorescence in situ hybridization analysis. IgH and MALT1 gene fusion pattern with the LSI IGH/MALT1 Dual Color, Dual Fusion Translocation Probe (Vysis, Downers Grove, IL, USA). One orange MALT1 signal (shown with the white arrowhead), one green IgH signal (shown with the black arrowhead), and two fusion IgH/MALT1 signals are present (shown with the yellow arrowhead).

**Figure 2:**
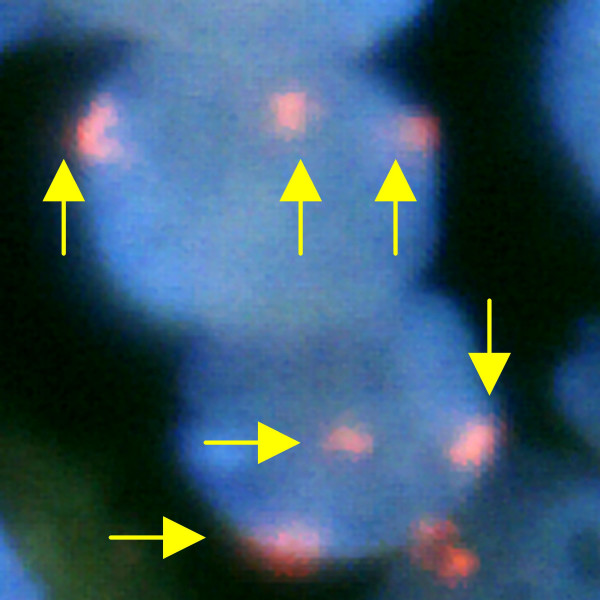
Representative result of fluorescence in situ hybridization analysis. Trisomy 18 is present as detected by the CEP 18 Orange Probe. Three orange signals are seen in one cell (shown with the yellow arrowhead).

The results of FISH analysis are shown in Table 2. We defined a case as having trisomy when three centromeres were recognized in the FISH analysis. Only one case was positive for t(14;18)(q32;q21) (Fig. 1). There were 21 (62%) cases with trisomy 3, 16 (47%) cases with trisomy 18 (Fig. 2), and 8 (24%) cases with both. There were two cases with trisomy 7 and no cases with trisomy 12. The one patient who showed extra MALT1 signals, which are suggestive of MALT1 gene splitting, was negative for t(11;18)(q21;q21). This case did not exhibit extra signals of centromere 18 or splitting of the MALT1 gene with the use of the MALT 1 breakapart probe, thus, MALT1 gene amplification was suggested.

The relationship between the clinical factors of the patients and the detection of trisomy 18 is shown in Table 3. Patients with trisomy 18 tended to be female, younger than 50 years old and most frequently showed involvement of the conjunctiva. None of the cases with trisomy 18 showed involvement of the lachrymal gland. Patients with trisomy 3 tended to be older than 50 years of age, however, no other significant correlation with the clinical features was observed (data not shown). There were also no significant correlations between other chromosomal aberrations and the clinical features.

**Figure 3:**
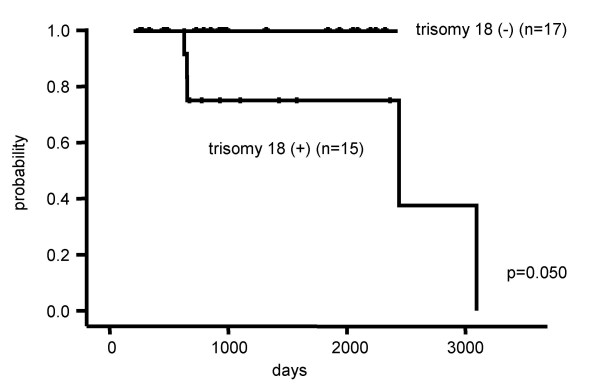
Time to recurrence in relation to the presence of trisomy 18.

**Figure 4:**
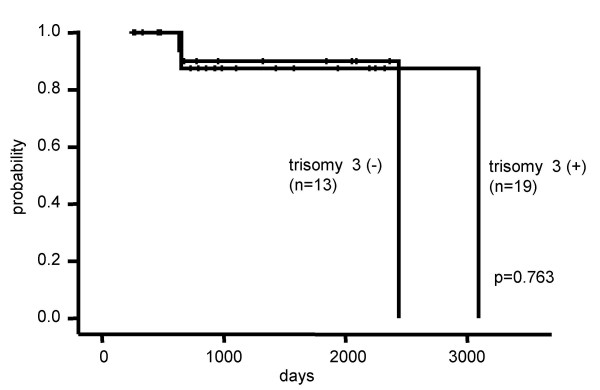
Time to recurrence in relation to the presence of trisomy 3.

The disease recurred in five cases, and all of these cases had trisomy 18. As shown in Fig. 3, there was a significant difference in the time to recurrence between patients with and without trisomy 18. On the other hand, as shown in Fig. 4, there was no significant difference in the time to recurrence between patients with and without trisomy 3. There were also no significant correlations between any of the other chromosomal aberrations and the time to recurrence (data not shown).

### Results of the histological review and the relationship among the clinical features, results of FISH analysis and the histopathological findings

Table 4 shows that the morphological features were heterogeneous, and the grades of the histopathological changes in the tumor varied. We tried to extract factors that might potentially influence the histopathological characteristics of the tumors: we found that the morphological characteristics were related to the site of involvement. Table 5 shows that cases with involvement of the conjunctiva were associated with specific morphological features: these tumors showed less nodularity (almost diffuse) and a greater abundance of polykaryocytes than tumors arising from other sites. Cases with lachrymal gland involvement showed more nodularity, a greater abundance of large lymphoid cells, and reactive T cells.

We attempted to identify the existence of any correlation between the cytogenetic heterogeneity as revealed by FISH analyses and the morphological features. Table 6 shows that tumors showing trisomy 18 tended to show less nodularity, plasma cells and large lymphoid cells, and a greater abundance of prominent LELs and mitotic cells, residual reactive germinal centers and monocytoid B cells as compared with tumors not showing trisomy 18. Among these parameters, significant correlations were observed between the cytogenetic characteristics and the degree of nodularity and abundance of LELs. There were no correlations between trisomy 3 or other chromosomal aberrations with the histopathological features (data not shown).

## Discussion

A number of karyotypic abnormalities have been reported in MALT lymphomas. Among these, t(11;18)(q21;q21) was the most frequently encountered structural chromosomal abnormality resulting from the fusion of the apoptosis inhibitor-2 (API2) gene and the MALT1 gene at the 11q21 and 18q21 breakpoints [9,20-22]. Streubel et al. indicated, based on the results of a FISH analysis, that t(14;18)(q32;q21) involving IGH and MALT1 was another frequently encountered chromosomal aberration in MALT lymphomas [22]. In their report, three out of eight cases (38%) of ocular adnexal MALT lymphoma had t(14;18)(q32;q21). Recently, the same group reported that t(3;14)(p14.1;q32) is also a recurrently observed chromosomal aberration in MALT lymphomas, especially those arising from the ocular adnexa; four out of 20 cases (20%) of ocular adnexal MALT lymphoma examined by them had this translocation [23].

The results of the FISH analysis revealed that translocations such as t(11;18)(q21;q21) and t(14;18)(q32;q21) were rather rare; only one case showed t(14;18)(q32;q21). In the study by Streubel et al., nine out of the 37 cases (24%) of ocular adnexal MALT lymphoma were positive for t(14;18)(q32;q21) and one out of the 37 (3%) cases were positive for t(11;18)(q21;q21) according to the results of FISH analysis [24]. This could be related to the geographic or genetic background; geographic heterogeneity has been shown for follicular lymphoma, another type of lymphoma; this tumor universally shows t(14;18)(q32;q21) in Europe and the US, but this is rare in this country [18]. Alternatively, it could merely reflect the small number of samples. More cases are needed to draw definitive conclusions.

In contrast, we found that aneuploidy was rather common. Approximately half of all the tumors showed trisomy 3 and/or 18. The frequency was at the upper end of the previously reported range of 10–50% [7,8,24]. This high frequency could be attributed to the methodology employed by us; we used the interphase FISH method, which can detect cytogenetic abnormalities at a greater frequency than conventional cytogenetic analysis. Since ocular adnexal MALT lymphoma is an indolent disease, interphase FISH analysis may be a more suitable method to detect abnormalities.

The ocular adnexal MALT lymphomas also exhibited morphologic heterogeneity, and the degree varied. Several reports revealed that the majority of ocular adnexal lymphomas were the MALT type. To the best of our knowledge, there have been no detailed analyses of the histopathological characteristics of ocular adnexal MALT lymphomas [12-16]. We suggest that since we analyzed many cases, significant morphological differences were found between cases with trisomy 18 and those without.

The differences in the morphological features of the tumors were related to the primary site of involvement of the MALT lymphomas; the number of large lymphoid cells, polykaryocytes and LELs, and the degree of nodularity of the tumor varied significantly depending on the site of origin of the tumors. It may only seem natural that tumors arising from different sites show different morphological characteristics; however, there have been no reports focusing on this issue among ocular adnexal MALT lymphomas, partly because of the small number of patients. The morphological differences suggest that MALT lymphomas of ocular adnexa origin might represent a combination of several diseases which may need to be analyzed separately.

The morphological features were also related to the chromosomal aberrations; more specifically speaking, the presence of trisomy 18 was associated with a specific morphology. The tumors in cases with trisomy 18 had more LELs and less nodularity than those without trisomy 18. In addition, we found that the cases with trisomy 18 had shorter recurrence-free survivals than those without trisomy 18. These five cases showed no significantly different characteristics from the other cases in terms of the sex or age of the patient, the site of involvement, laterality, initial therapy, stage, or the serum LDH levels at the time of initial presentation. Trisomy 18 was strongly related to the recurrence-free survival rate. These results show that tumors with trisomy 18 may represent a characteristic and one unique subgroup among primary ocular adnexal MALT lymphomas. This recognition might compare well with the situation of gastric MALT lymphomas with t(11;18)(q21;q21), which constitutes a specific subgroup in terms of the clinical setting, patient prognosis, and morphological findings among all cases of gastric MALT lymphoma [25,26]. The biological significance of these specific features is unclear and further gene expression profiling and/or competitive genomic hybridization assays are necessary.

Recently, it has been reported that some ocular lymphomas may be related to Chlamydia and hepatitis C virus infection, and the involvement of these agents might be the reason for the heterogeneity among the tumors [27-29]. In our series, we did not conduct these analyses, and the relation between infection with these agents and the existence of trisomy 18 is currently unknown.

## Conclusion

We detected aneuploidy in cases of ocular adnexal MALT lymphomas, and also morphological heterogeneity. These results demonstrate that ocular adnexal MALT lymphomas differ in characteristics from those at other anatomic sites, and also show significant heterogeneity. Also, we suggest that these tumors showing trisomy 18 might represent a unique entity.

## Competing interests

The author(s) declare that they have no competing interests.

## Authors' contributions

KT, NS, YY and YK carried out the FISH analysis. KT, AMM and YM carried out the histological analysis. KT, NS, AK, TW and KT participated in the design of the study and performed the statistical analysis. KT, NS, MH and YK conceived of the study, and participated in its design and coordination and helped to draft the manuscript. All authors read and approved the final manuscript.

## Pre-publication history

The pre-publication history for this paper can be accessed here:



## Figures and Tables

**Table 1:** Patient characteristics

No. of patients (n = 34)	
Sex	
Male/Female	23/11
	
Age (years)	
Median/Range	57/15–90
	
Site of involvement	
Conjunctiva	9
Orbit	21
Lachrymal gland	4
	
Laterality	
Right	13
Left	13
Bilateral	8
	
Stage	
I, II	31
III, IV	3
	
LDH>normal	5
	
Initial therapy*	
Radiation	21
Chemotherapy	3
Chemotherapy + Radiation	3
Observation	5

* Two patients lost to follow-up after the diagnosis.

**Table 2:** Results of fluorescence in situ hybridization analysis

No. of Patients (%) (n = 34)	
t(11;18)(q21;q21)	0 (0%)
t(14;18)(q32;q21)	1(3%)
trisomy 18*	16 (47%)
trisomy 3**	21 (62%)
trisomy 7	2 (6%)
trisomy 12	0 (0%)
MALT 1 gene amplification	1 (3%)
Normal	3 (9%)

* There were eight cases with trisomy 18 alone and eight cases with trisomy 18 and trisomy 3.

** There were eleven cases with trisomy 3 alone, eight with trisomy 3 and   trisomy 18, one with t(14;18)(q32;q21), and one with MALT1 gene   amplification.

**Table 3:** Relationship between clinical features and the presence of trisomy 18 as revealed by fluorescence in situ hybridization

	No. of patients		
	
clinical factor	trisomy18 (+) (n = 16)	trisomy 18 (-) (n = 18)	p-value
Sex			
male	9	14	0.166
female	7	4	
			
Age			
< 50	6	2	0.080
≥ 50	10	16	
			
Site of involvement			
orbit	10	11	0.099
conjunctiva	6	3	
lachrymal grand	0	4	
			
Laterality			
unilateral	12	14	0.583
bilateral	4	4	
			
Stage			
I	16	15	0.136
II-IV	0	3	
			
LDH			
normal	14	15	0.559
> normal	2	3	

**Table 4:** Results of the grades of the histopathological changes in all 34 cases

histopathological features		No. of patients		
		
	-	+	2+	3+
plasma cells	5	21	7	1
Dutcher bodies	21	11	2	NA
monocytoid B cells	6	16	11	1
residual reactive germinal centers	7	13	14	NA
large lymphoid cells	2	24	8	NA
polykaryocytes	12	15	7	NA
brown-pigment-laden histiocytes	17	10	7	NA
mitotic cells	4	28	2	NA
nodularity	7	14	13	NA
lymphoepithelial lesions*	4	7	2	NA
reactive T cells**	0	22	10	0

NA: not applicable

* 21 cases were not applicable.

** Two cases were not done.

**Table 5:** Relationship between histopathological findings and the site of involvement

No. of patients					No. of patients				
	conjunctiva	orbit	lachrymal grand	p-value		conjunctiva	orbit	lachrymal grand	p-value

plasma cells					brown-pigment-laden histiocytes				
2+, 3+	1	5	2		(-)	5	8	4	
2+ <	8	16	2	0.347	(+)	4	13	0	0.074
Dutcher bodies					mitotic cells				
(-)	5	12	4		(-)	1	2	1	
(+)	4	9	0	0.305	(+)	8	19	3	0.742
monocytoid B cells					nodularity				
(-)	2	4	0		(-)	5	2	0	
(+)	7	17	4	0.697	(+)	4	19	4	0.017
residual reactive germinal centers					lymphoepithelial lesions *				
(-)	2	4	1		(-)	0	1	3	
(+)	7	17	3	1.000	(+)	6	2	1	0.053
large lymphoid cells					reactive T cells **				
2+	1	4	3		1+	8	14	0	
(-), 1+	8	17	1	0.050	2+	1	5	4	0.005
polykaryocytes									
(-)	0	9	3						
(+)	9	12	1	0.011					

* 21 cases were not applicable.

** Two cases were not done.

**Table 6:** Relationship between the histopathological grades and the presence of trisomy 18 as revealed by fluorescence in situ hybridization

	No. of patients				No. of patients		
	trisomy 18 (+)	trisomy 18 (-)	p-value		trisomy 18 (+)	trisomy 18 (-)	p-value

plasma cells				brown-pigment-laden histiocytes			
2+, 3+	2	6	0.153	(-)	7	10	0.366
2+ <	14	12		(+)	9	8	
Dutcher bodies				mitotic cells			
(-)	10	11	0.607	(-)	0	4	0.066
(+)	6	7		(+)	16	14	
monocytoid B cells				nodularity			
(-)	1	5	0.116	(-)	6	1	0.029
(+)	15	13		(+)	10	17	
residual reactive germinal centers				lymphoepithelial lesions *			
(-)	1	6	0.061	(-)	0	4	0.049
(+)	15	12		(+)	6	3	
large lymphoid cells				reactive T cells **			
2+	2	6	0.153	1+	12	10	0.183
(-), 1+	14	12		2+	3	7	
polykaryocytes							
(-)	5	7	0.459				
(+)	11	11					

* 21 cases (10 trisomy 18 (+), 11 trisomy 18 (-)) were not applicable.

** Two cases (one trisomy 18 (+), one trisomy 18 (-)) were not done.
